# Alteration of Blood Lactate Levels in Severe Falciparum Malaria: A Systematic Review and Meta-Analysis

**DOI:** 10.3390/biology10111085

**Published:** 2021-10-22

**Authors:** Polrat Wilairatana, Wanida Mala, Manas Kotepui, Kwuntida Uthaisar Kotepui

**Affiliations:** 1 Department of Clinical Tropical Medicine, Faculty of Tropical Medicine, Mahidol University, Bangkok 10400, Thailand; polrat.wil@mahidol.ac.th; 2 Medical Technology, School of Allied Health Sciences, Walailak University, Tha Sala, Nakhon Si Thammarat 80160, Thailand; wanida.ma@wu.ac.th (W.M.); manas.ko@wu.ac.th (M.K.)

**Keywords:** lactate, lactic acid, blood, falciparum, malaria

## Abstract

**Simple Summary:**

Alteration of blood lactate levels in patients with severe falciparum malaria is well recognized. However, data on blood lactate in literatures were based on a limited number of participants. The present systematic review aimed to collate the blood lactate levels recorded in the literature and used a metaanalysis approach to pool the evidence in a larger sample size than that used in the individual studies to determine the trend. Results from this study will provide the pooled evidence of blood lactate levels in patients with severe malaria for further studies that identifying patients with a high risk of developing severe malaria or death.

**Abstract:**

Metabolic acidosis in severe malaria usually occurs in the form of lactic acidosis. The present study aimed to collate articles from the literature that have reported blood lactate levels in patients with severe malaria and tested the hypothesis that blood lactate levels are elevated in patients with malaria compared to those with uncomplicated malaria. Moreover, the difference in lactate levels between patients who died and those who survived was estimated using a meta-analytic approach. Potentially relevant studies were searched for in PubMed, Web of Science, and Scopus. The quality of the included studies was assessed using the Jadad scale and strengthening the reporting of observational studies in epidemiology (STROBE). The pooled mean blood lactate in patients with severe malaria, the pooled weighted mean difference (WMD) of blood lactate between patients with severe malaria and those with uncomplicated malaria, and the pooled WMD and 95% CI of blood lactate between patients who died from and those who survived severe malaria were estimated using the random-effects model. Heterogeneity among the outcomes of the included studies was assessed using Cochran’s Q and I^2^ statistics. A meta-regression analysis was performed to identify the source(s) of heterogeneity of outcomes among the included studies. A subgroup analysis was further performed to separately analyze the outcomes stratified by the probable source(s) of heterogeneity. Publication bias was assessed by the visual inspection of the funnel plot asymmetry. Of 793 studies retrieved from the searches, 30 studies were included in qualitative and quantitative syntheses. The pooled mean lactate in patients with severe malaria was 5.04 mM (95% CI: 4.44–5.64; I^2^: 99.9%; *n* = 30,202 cases from 30 studies). The mean lactate in patients with severe malaria (1568 cases) was higher than in those with uncomplicated malaria (1693 cases) (*p* = 0.003; MD: 2.46; 95% CI: 0.85–4.07; I^2^: 100%; nine studies). The mean lactate in patients with severe malaria who died (272 cases) was higher than in those with severe malaria who survived (1370 cases) (*p* < 0.001; MD: 2.74; 95% CI: 1.74–3.75; I^2^: 95.8%; six studies). In conclusion, the present study showed a high mean difference in blood lactate level between patients with severe malaria and patients with uncomplicated malaria. In addition, there was a high mean difference in blood lactate level between patients with severe malaria who died compared to those with severe malaria who survived. Further studies are needed to investigate the prognostic value of blood lactate levels to identify patients who are at high risk of developing severe malaria or dying.

## 1. Introduction

Malaria in humans is caused by one of six *Plasmodium* species: *P. falciparum*, *P. vivax*, *P. malariae*, *P. ovale curtisi*, *P. ovale wallikeri*, and *P. Knowlesi* [1,2]. Although some non-*P. falciparum* species may cause severe malaria [2,3,4,5], *P*. *falciparum* is still the leading cause of severe malaria among children in Africa and adults in non-endemic countries [6,7]. Severe malaria is defined as the presence of *P. falciparum* with one of the following criteria: impaired consciousness, prostration, multiple convulsions, acidosis, hypoglycemia, severe malarial anemia, renal impairment, jaundice, pulmonary edema, significant bleeding, shock, or hyperparasitemia [8]. Among the potentially severe complications, metabolic acidosis is one of the strongest predictors of mortality in patients with severe malaria [9,10,11]. Metabolic acidosis in severe malaria usually occurs in the form of lactic acidosis; high levels of lactic acid will produce anion gap metabolic acidosis [8]. However, the etiology of lactic acidosis in severe malaria is poorly understood. The following mechanisms have been proposed: the increased production of lactate by malaria parasites, parasite sequestration, anemia, circulatory failure, immune responses, and impaired lactate clearance by the liver or renal system [12].

Previous studies have also shown the occurrence of hyperlactatemia in severe falciparum malaria [13,14,15,16], as well as a higher lactate level in severe malaria than in uncomplicated malaria [17,18,19]. Further, a higher lactate level was reported in patients with severe malaria who died than in those who survived [10,20,21]. A recent study on lactate-related mortality in severely ill febrile children in East Africa showed that hyperlactatemia was a strong risk factor for death at 72 h, while increased chance of survival was related to the clearance of lactate within 8 h [22]. Although information on blood lactate in patients with severe malaria or patients who died from malaria has been reported in several studies, data on blood lactate in these studies were based on a limited number of participants. Therefore, in the present study, we used a systematic review to collate the blood lactate levels recorded in the literature and used a meta-analysis approach to pool the evidence in a larger sample size than that used in the individual studies to determine the trend. In the present study, we aimed to test the hypothesis that blood lactate levels are elevated in patients with complicated malaria compared to those with uncomplicated malaria. Moreover, the difference in lactate levels between patients who died and those who survived was estimated using a meta-analysis approach.

## 2. Method

### 2.1. Protocol and Registration

For this systematic review, we followed the Preferred Reporting Items for Systematic Reviews and Meta-Analyses (PRISMA) [23] guidelines. The systematic review was registered at PROSPERO with a registered ID: CRD42021276043.

### 2.2. Eligibility Criteria

The inclusion criteria were all studies that reported the following information: (1) severe falciparum malaria, (2) mean blood lactate at admission (baseline data before treatment or intervention was given). The exclusion criteria were studies for which any of the following applied: (1) did not feature cases of severe malaria, (2) were performed in the same groups of participants, (3) measured lactate after participants were given the treatment or intervention, (4) data on blood lactate could not be extracted, (5) were short reports or studies with less than 10 participants, (6) were review articles, (7) were case reports/case series, or (8) were in vitro or in vivo studies.

### 2.3. Information Sources and Search Strategy

Potentially relevant studies were searched for using PubMed, Web of Science, and Scopus between 7 and 9 August 2021 without any restrictions regarding the publication date. The following search strategy was used to retrieve the relevant studies “(lactate OR “lactic acid”) AND (malaria OR plasmodium) AND (severe OR complicated)”. The search terms “lactate”, “malaria”, and “severe” were cross-checked with the Medical Subject Headings (MeSH) to assure that the terms used were appropriate (Table S1). Additional searches were performed by reviewing reference lists of the included studies and Google Scholar to assure that relevant studies were not missed during the searches.

### 2.4. Study Selection

Two authors (MK and KUK) were responsible for study selection (independently) based on the eligibility criteria. First, duplicates were removed before study selection. Second, the titles and abstracts of the studies were screened, and non-relevant studies were excluded. Third, the full texts of the studies were examined; studies that did not meet the eligibility criteria were excluded, and reasons were given for their exclusion. Any discrepancy in study selection between the two authors was resolved by discussion until a consensus was reached.

### 2.5. Data Collection Process

Data extraction from studies that met the eligibility criteria was performed independently by the two authors (MK and KUK). The following information was extracted: the name of the first author, study site, study year, study design, types and numbers of participants enrolled, mean age of the participants (years), age range (years), percentage of males, mean and standard deviation of blood lactate (mmol/µL) at admission (baseline data), and mean parasitemia level (cells/µL).

### 2.6. Outcomes

The primary outcome was the pooled mean blood lactate in patients with severe malaria. The secondary outcome was the pooled weighted mean difference (WMD) and 95% confidence interval (CI) of blood lactate between patients with severe malaria and those with uncomplicated malaria. The tertiary outcome was the pooled WMD and 95% CI of blood lactate, allowing a comparison between patients who died from and those who survived severe malaria. 

### 2.7. Quality of the Included Studies

The quality of the included studies was assessed by two authors (MK, WM) using the Jadad scale for reporting randomized controlled trials [24] and the strengthening of the reporting of observational studies in epidemiology (STROBE) [25] guidelines. For the Jadad scale, studies with scores of 0–3 were determined to be of poor quality, while studies with scores of 4–8 were determined to be of good to excellent quality (high quality), as described elsewhere [26]. For STROBE, the studies were categorized as either high quality (over 75% of the STROBE checklist) or low quality (under 75% of the STROBE checklist), as described elsewhere [27].

### 2.8. Synthesis of Results

The pooled mean blood lactate in patients with severe malaria, the pooled WMD of blood lactate between patients with severe malaria and those with uncomplicated malaria, and the pooled WMD and 95% CI of blood lactate for patients who died from and those who survived severe malaria were estimated using the random-effects model. The medians and ranges of blood lactate, age, and parasitemia level in the included studies were transformed to means and standard deviations, as described previously [28]. For studies that reported the mean blood lactate value without the standard deviation, we borrowed a value for the standard deviation from another study with a similar mean [29]. Heterogeneity among the outcomes of the included studies was assessed using Cochran’s Q (p less than 0.05 indicated significant heterogeneity) and I^2^ statistics (I^2^ < 25% indicated a low heterogeneity, a value between 25% and 50% indicated moderate heterogeneity, and a value of over 50% indicated a high heterogeneity). A meta-regression analysis was performed to identify the source(s) of heterogeneity of the outcome among the included studies. A subgroup analysis was performed to separately analyze the outcomes stratified by the probable source(s) of heterogeneity. Publication bias was assessed by the visual inspection of the funnel plot asymmetry. If the funnel plot was determined to be asymmetrical, the contour-enhanced funnel plot was inspected to identify the source of the asymmetry. Egger’s test was also performed to test whether the pooled estimates were affected by the small study effect. All statistical analyses were performed with Stata version 14.0 (StataCorp, College Station, TX, USA).

## 3. Results

### 3.1. Search Results

**Figure 1:**
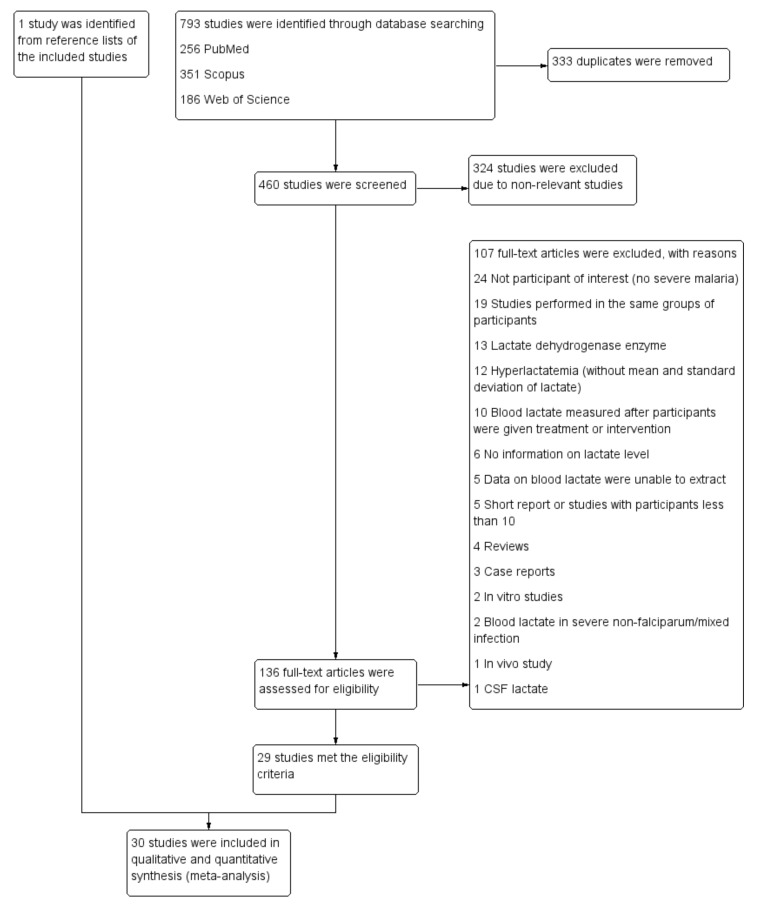
Study flow diagram. Abbreviation: CSF, cerebrospinal fluid.

Seven hundred and ninety-three studies were retrieved from PubMed (256 studies), Scopus (351 studies), and Web of Science (186 studies). After 333 duplicates were removed, the titles and abstracts of 460 studies were screened, and 324 non-relevant studies were removed. One hundred and thirty-six studies remained for a full-text examination. A total of 107 studies did not meet the eligibility criteria and were excluded for various reasons; 24 did not feature participants of interest (no severe malaria), 19 studies were performed in the same groups of participants, 13 focused on the lactate dehydrogenase enzyme, 12 focused on hyperlactatemia (without the mean and standard deviation of lactate), 10 only measured blood lactate after participants were given the treatment or intervention, 6 provided no information on the lactate level, in 5 we were unable to extract data on blood lactate, 5 were short reports or studies with less than 10 participants, 4 were reviews, 3 were case reports, 2 were in vitro studies, 2 focused on blood lactate in severe non-falciparum/mixed infection, 1 was an in vivo study, and 1 focused on CSF lactate. From this, 29 studies [17,18,19,20,21,30,31,32,33,34,35,36,37,38,39,40,41,42,43,44,45,46,47,48,49,50,51,52,53] were determined to meet the eligibility criteria and were included in the present study. An additional study [10] was identified from reviewing the reference lists of the included studies. Finally, 30 studies [10,17,18,19,20,21,30,31,32,33,34,35,36,37,38,39,40,41,42,43,44,45,46,47,48,49,50,51,52,53] were included in our qualitative and quantitative syntheses (Figure 1).

### 3.2. Characteristics of the Included Studies

The studies included in the present review were published between the years 1988 and 2020 (Table 1). Most of the included studies were conducted in Africa (73.3%, 22/30), while fewer studies were conducted in Asia (23.3%, 7/30) and Europe (Netherlands, 1 study). Among the studies conducted in Africa, studies were conducted in Uganda (18.2%, 4/22) [19,34,44,45], Malawi (18.2%, 4/22) [39,41,43,48], Kenya (18.2%, 4/22) [32,33,36,47], Gambia (18.2%, 4/22) [35,40,49,51], Ghana (9.09%, 2/22) [17,31], Gabon (9.09%, 2/22) [38,46], and Cameroon [37], and one study was conducted in four countries (Gambia, Malawi, Gabon, and Kenya) [30]. Among the studies conducted in Asia, studies were conducted in Bangladesh (28.6%, 2/7) [20,21], Thailand (28.6%, 2/7) [42,52], Indonesia [53], Malaysia [18], and Vietnam [10]. Most of the included papers (80%, 24/30) were cohort studies, while four were case-control studies [17,35,39,47], and two studies were clinical trials [31,52]. Most of the included studies enrolled children with severe malaria (53.3%, 16/30) [30,31,32,33,34,36,38,39,40,41,44,45,47,48,49,51], adults with severe malaria (16.7%, 5/30) [10,21,42,52,53], children with uncomplicated and severe malaria (16.67%, 5/30) [19,35,37,43,46], adults with uncomplicated and severe malaria (6.67%, 2/30) [20,50], and children with severe and uncomplicated malaria (6.67%, 2/30) [17,18]. Other characteristics of the included studies are shown in Table 1.

### 3.3. Quality of the Included Studies

Twenty-five studies [10,18,19,20,21,30,31,32,33,34,35,37,38,39,40,41,42,44,45,47,49,50,51,52,53] were determined to be high-quality studies, whereas five [17,36,43,46,48] were determined to be low-quality studies (Table S2). The low-quality studies were retained for a qualitative synthesis.

### 3.4. Mean Lactate Level in Severe Malaria

**Figure 2:**
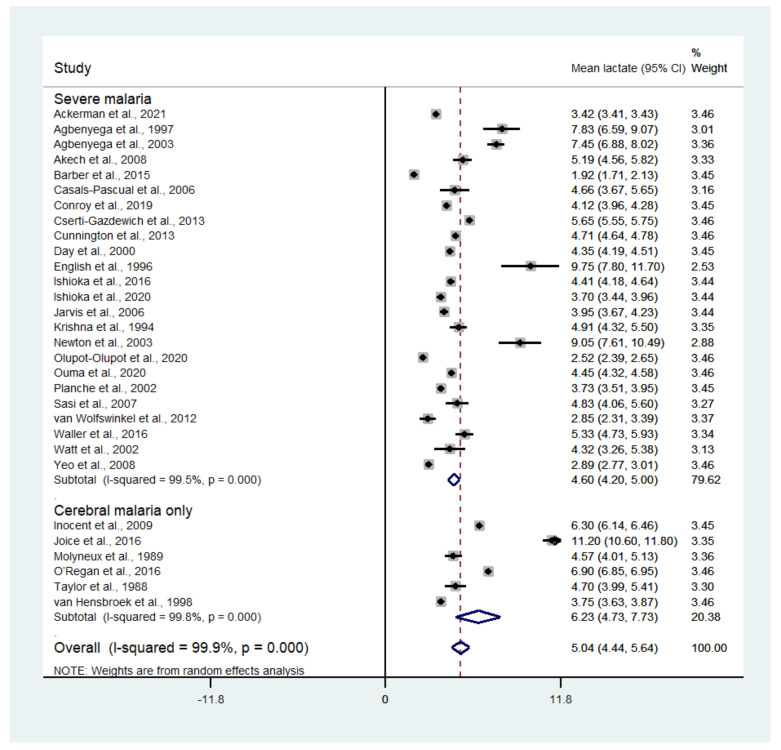
Mean lactate level in patients with severe malaria. Abbreviation, CI: confidence interval.

The pooled mean lactate in patients with severe malaria was estimated using the random-effects model. Results showed that the pooled mean lactate level in patients with severe malaria was 5.04 mM (95% CI: 4.44–5.64, I^2^: 99.9%, *n* = 30,202 cases from 30 studies). A subgroup analysis of the types of complications seen (cerebral malaria only and any types of severe complications) showed that the pooled mean lactate level in patients with cerebral malaria was 6.23 mM (95% CI: 4.73–7.73, I^2^: 99.8%, *n* = 921 cases from six studies), while the pooled mean lactate level in patients with any severe complications was 4.60 mM (95% CI: 4.20–5.00, I^2^: 99.5%, *n* = 29,281 cases from 24 studies) (Figure 2). A meta-regression analysis showed that the mean age, mean parasitemia, or proportion of males in the studies did not confound the pooled mean estimates (*p* > 0.05).

**Figure 3:**
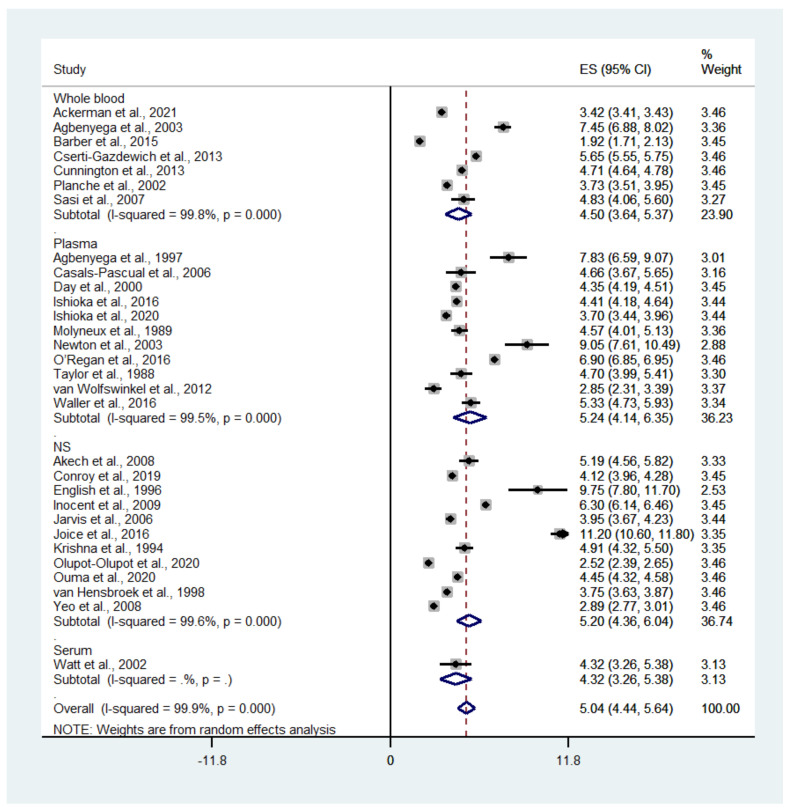
Mean lactate level in patients with severe malaria according to the type of blood sample used. Abbreviations: ES, estimated mean lactate; CI: confidence interval; NS, not specified.

A subgroup analysis of the different types of blood samples (whole blood or plasma) showed that the pooled mean whole blood lactate level in patients with severe malaria was 4.5 mM (95% CI: 3.64–5.37, I^2^: 99.8%, *n* = 27,163 cases from seven studies), while the pooled mean plasma lactate level in patients with severe malaria was 5.24 mM (95% CI: 4.14–6.35, I^2^: 99.5%, *n* = 1177 cases from 11 studies) (Figure 3).

**Figure 4:**
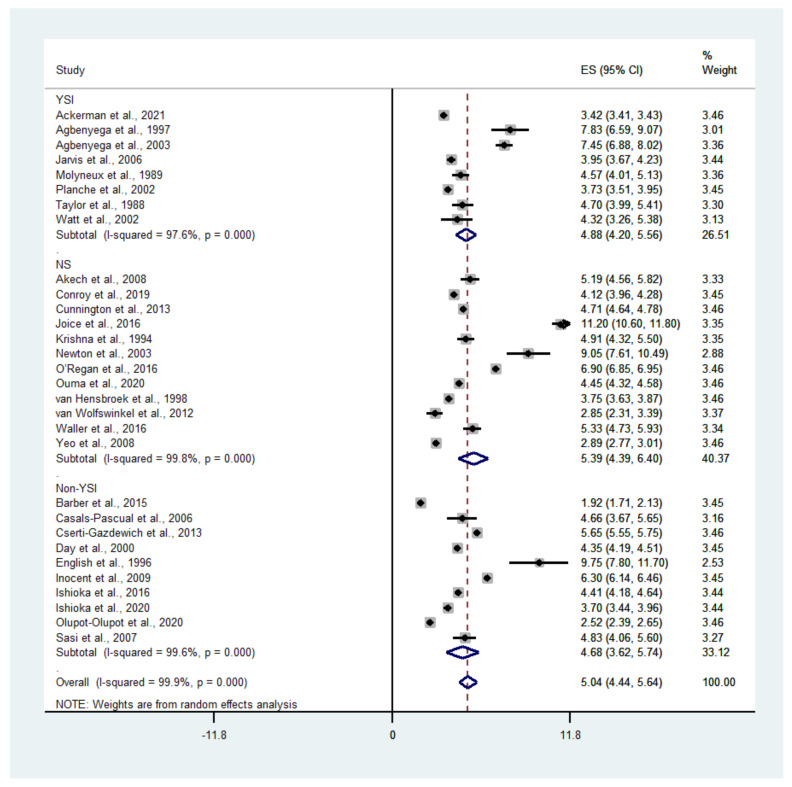
Mean lactate level in patients with severe malaria determined by different analyzers. Abbreviations: ES, estimated mean lactate; CI, confidence interval; NS, not specified.

A subgroup analysis of the blood lactate level determined using an analyzer (YSI and non-YSI) showed that the pooled mean lactate level in patients with severe malaria according to a YSI analyzer was 4.88 mM (95% CI: 4.20–5.56, I^2^: 97.6%, *n* = 26,457 cases from eight studies), while the pooled mean lactate level in patients with severe malaria according to a non-YSI analyzer was 4.68 mM (95% CI: 3.62–5.74, I^2^: 99.7%, *n* = 1578 cases from 10 studies) (Figure 4).

### 3.5. Mean Lactate Level in Patients with Severe Malaria Who Died

**Figure 5:**
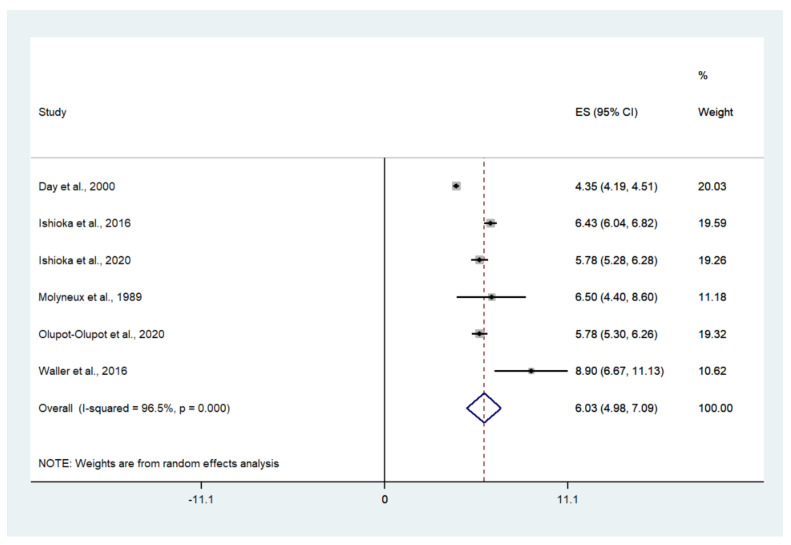
Mean lactate level in patients with severe malaria who died. Abbreviations: ES, estimated mean lactate; CI: confidence interval; NS, not specified.

The pooled mean lactate level in patients with severe malaria who died was estimated using the random-effects model. The results showed that the pooled mean lactate level in patients with severe malaria who died was 6.03 mM (95% CI: 4.98–7.09, I^2^: 96.5%, *n* = 272 cases from six studies) (Figure 5).

### 3.6. The Difference in Mean Lactate Level between Severe and Uncomplicated Malaria

**Figure 6:**
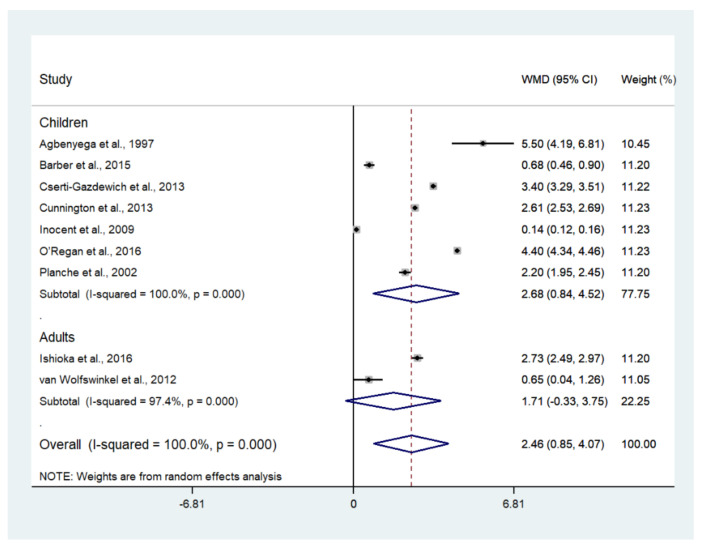
The difference in mean lactate level between patients with severe and uncomplicated malaria. Abbreviations: WMD, weighted mean difference; CI, confidence interval.

**Figure 7:**
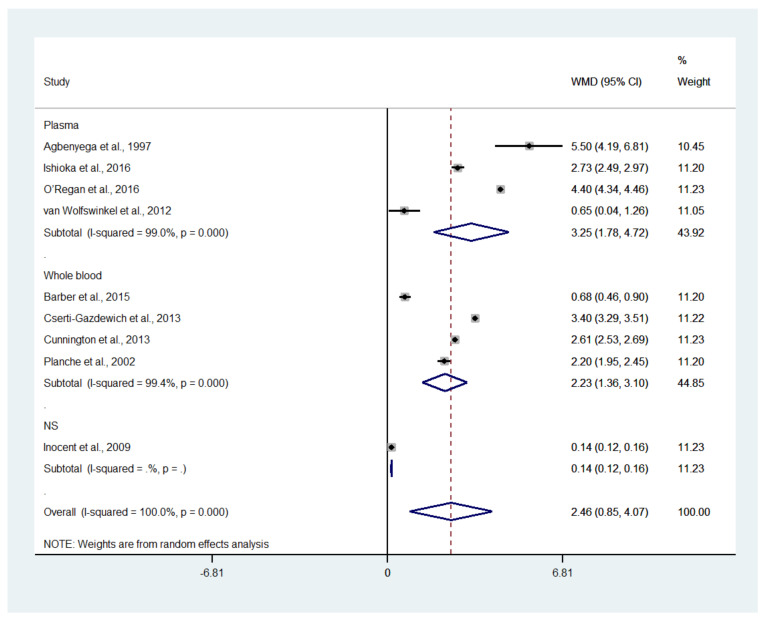
The difference in mean lactate level between patients with severe and uncomplicated malaria according to the different types of blood samples. Abbreviations: WMD, weighted mean difference; CI, confidence interval.

**Figure 8:**
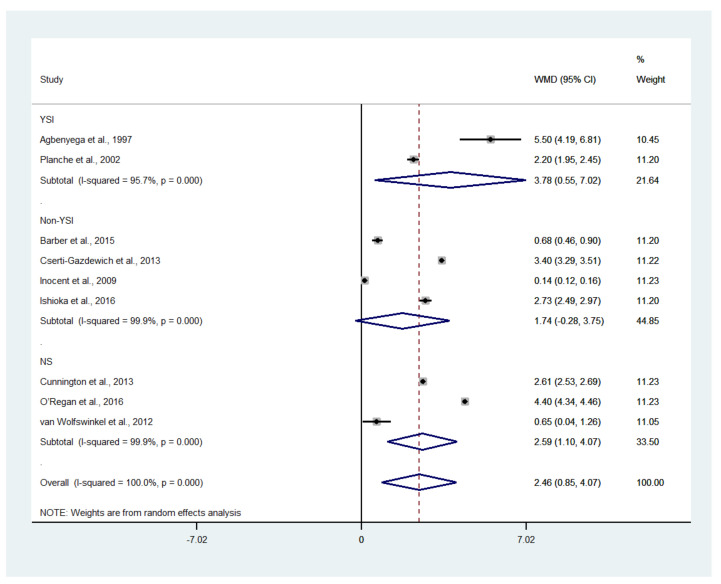
The difference in mean lactate level between patients with severe and uncomplicated malaria according to analyzers. Abbreviations: WMD, weighted mean difference; CI, confidence interval.

**Figure 9:**
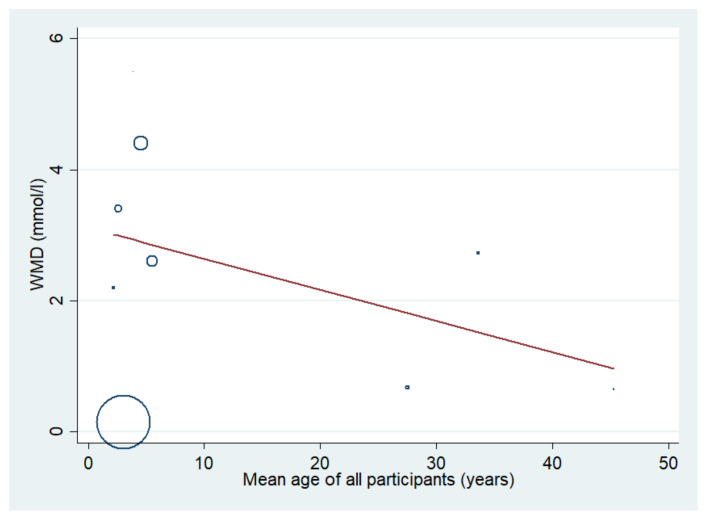
The difference in mean lactate level between patients with severe and uncomplicated malaria using mean age as a covariate. Circles and dots show the WMD of individual studies. Abbreviations: WMD, weighted mean difference; CI, confidence interval.

The difference in the mean lactate level between patients with severe and uncomplicated malaria was estimated using the data available from nine studies [17,18,19,20,35,37,43,46,50]. The results showed that the mean lactate level in patients with severe malaria (1568 cases) was higher than in those with uncomplicated malaria (1693 cases) (*p* = 0.003; MD: 2.46; 95% CI: 0.85–4.07; I^2^: 100%; nine studies). However, subgroup analyses showed that the mean lactate level in patients with severe malaria (1319 cases) was higher than in those with uncomplicated malaria (1515 cases) in children (*p* = 0.004; MD: 2.68; 95% CI: 0.84–4.52; I^2^: 100%; seven studies), while no difference in mean lactate was found between two groups of adults (*p* = 0.100; MD: 1.71; 95% CI: −0.33–3.75; I^2^: 97.4%; severe malaria: 249 cases; 178 cases of uncomplicated malaria; two studies) (Figure 6). A subgroup analysis of the different types of blood samples showed that the mean lactate level in patients with severe malaria (438 cases) was higher than in those with uncomplicated malaria (246 cases) in studies measuring plasma lactate (*p* < 0.001; MD: 3.25; 95% CI: 1.78–4.72; I^2^: 99%; four studies), while the mean lactate in patients with severe malaria (1085 cases) was higher than in those with uncomplicated malaria (1353 cases) in studies measuring whole-blood lactate (*p* < 0.001; MD: 2.23; 95% CI: 1.36–3.10; I^2^: 99.4%; four studies) (Figure 7). A subgroup analysis of the analyzers showed that the mean lactate level in patients with severe malaria (140 cases) was higher than in those with uncomplicated malaria (39 cases) according to the YSI analyzer (*p* = 0.022; MD: 3.78; 95% CI: 0.55–7.02; I^2^: 95.7%; two studies), while the mean lactate level in patients with severe malaria (1141 cases) was higher than in those with uncomplicated malaria (1317 cases) according to the non-YSI analyzers (*p* = 0.091; MD: 1.74; 95% CI: −0.28–3.75; I^2^: 99.9%; two studies) (Figure 8). A meta-regression analysis showed that the mean age, mean parasitemia, and proportion of males in the studies did not confound the MD of the lactate between two groups (*p* > 0.05). For the meta-analysis using mean age as a co-variate, the MD of lactate seemed to become continuously lower with increasing age (Figure 9).

### 3.7. Lactate in Patients with Severe Malaria Who Died and Survived

**Figure 10:**
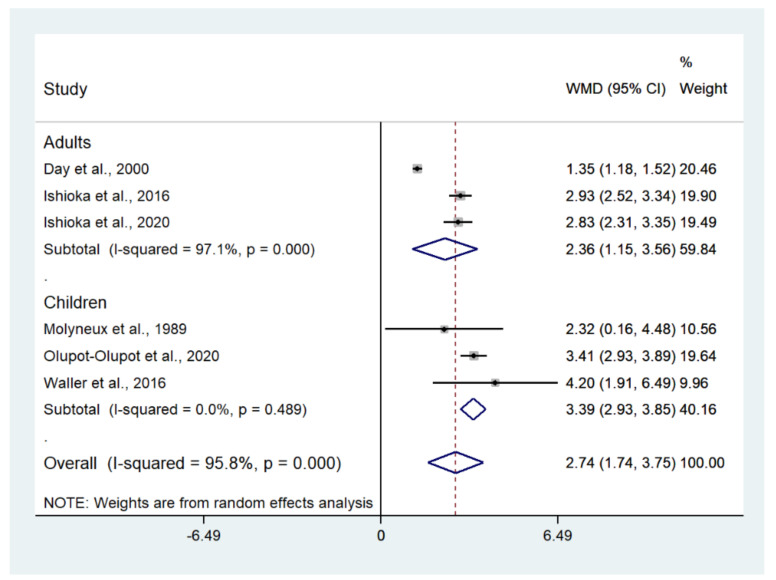
Lactate levels in patients with severe malaria who died and those who survived. Abbreviations: WMD, weighted mean difference; CI, confidence interval.

**Figure 11:**
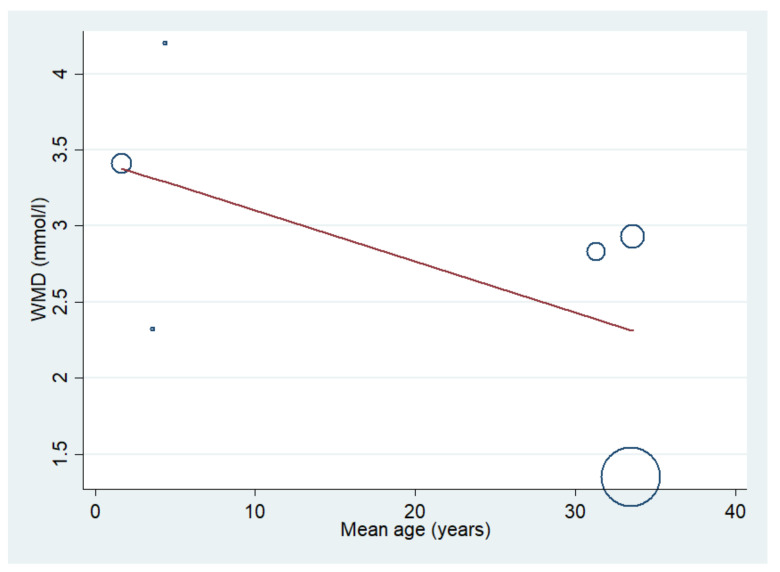
Lactate levels in patients with severe malaria who died and those who survived using mean age as a covariate. Circles and dots show the WMD of individual studies. Abbreviations: WMD, weighted mean difference; CI, confidence interval.

**Figure 12:**
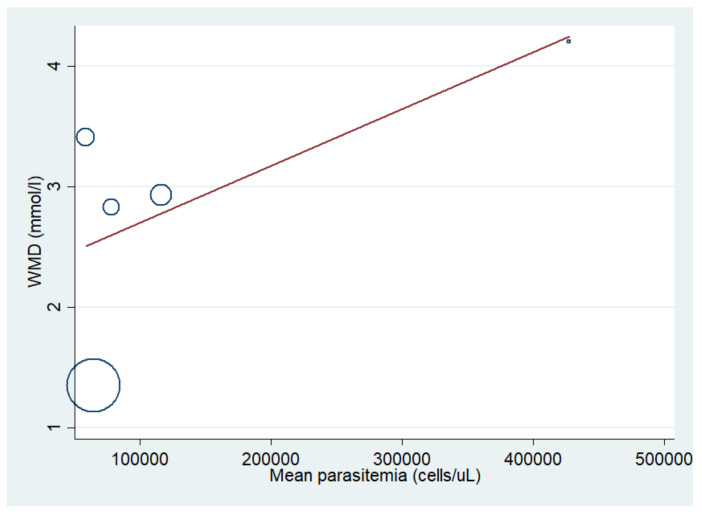
Lactate levels in patients with severe malaria who died and those who survived using mean parasitemia as a co-variate. Circles and dots show the WMD of individual studies. Abbreviations: WMD, weighted mean difference; CI, confidence interval.

The difference in mean lactate level between patients with severe malaria who died and those who survived was estimated using the data available from six studies [10,20,21,41,44,51]. The results showed that the mean lactate level in patients with severe malaria who died (272 cases) was higher than in those with severe malaria who survived (1370 cases) (*p* < 0.001; MD: 2.74; 95% CI: 1.74–3.75; I^2^: 95.8%; six studies). A subgroup analysis of the participants showed that the mean lactate level in patients with severe malaria who died (162 cases) was higher than in those with severe malaria who survived (559 cases) in adults (*p* < 0.001; MD: 2.36; 95% CI: 1.15–3.56; I^2^: 97.1%; three studies) and children (*p* < 0.001; MD: 3.39; 95% CI: 2.93–3.85; I^2^: 95.8%; 110 deaths and 811 survivals; three studies) (Figure 10). The meta-regression analysis showed that the mean age, mean parasitemia, and proportion of males in the studies did not confound the MD of lactate between the two groups (*p* > 0.05). For the meta-analysis using mean age as a co-variate, the MD of lactate seemed to become continuously lower with increasing age (Figure 11). For the meta-analysis using mean parasitemia as a co-variate, the MD of lactate seemed to become continuously higher with increasing age (Figure 12).

### 3.8. Publication Bias

**Figure 13:**
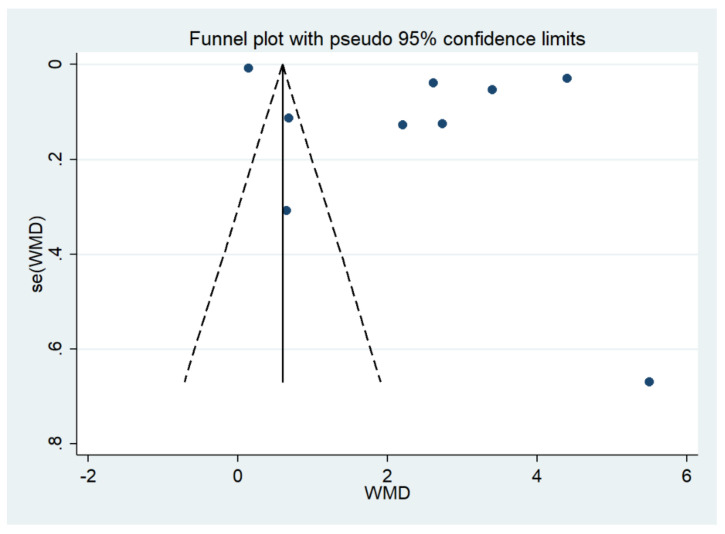
The funnel plot demonstrated an asymmetrical distribution of the estimates (blue dot) from the middle line. Abbreviations: se, standard error; WMD, weighted mean difference.

**Figure 14:**
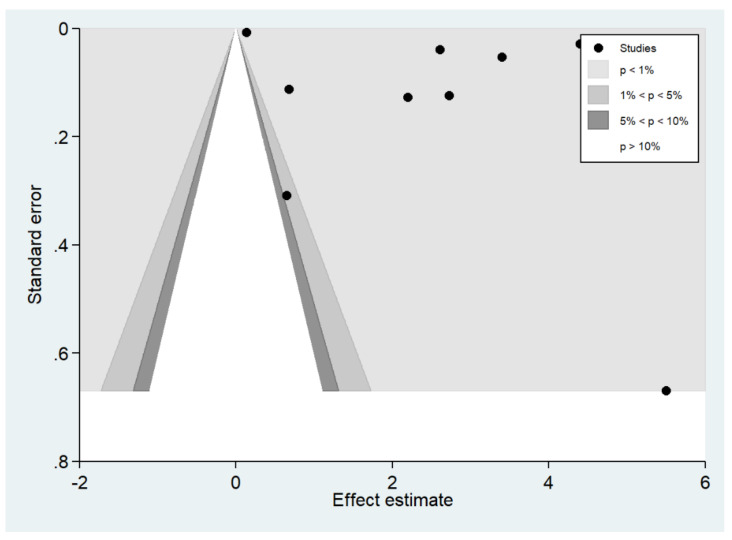
The contour-enhanced funnel plot demonstrated that the estimates (black dot) were located in the significant area (*p* < 0.1).

The funnel plot demonstrated an asymmetrical distribution of the estimates from the middle line (Figure 13). Egger’s test demonstrated that no small-study effects were found (*p* > 0.05). The contour-enhanced funnel plot demonstrated that the estimates were located in the significant area (*p* < 0.1), indicating that the funnel plot asymmetry was caused by heterogeneity in the outcomes of the included studies (Figure 14).

## 4. Discussion

The present study showed a high mean lactate level in patients with severe malaria (5.04 mM) with a high heterogeneity among the included studies. The high heterogeneity of the lactate level was due to the heterogeneity of the study sites, as some studies were conducted in Africa and some studies were conducted in Asia. In addition, the difference in the study designs and enrolled participants might have contributed to the high heterogeneity of the mean blood lead levels among patients with severe malaria. For example, the studies conducted in Kenya [36], Thailand [42], and Malawi [39] reported high mean lactate levels of between 9.05 mM and 11.20 mM. However, lower mean lactate levels between 1.92 mM and 8.89 mM were reported in studies conducted in Malaysia [18], Uganda [44], the Netherlands [50], and Indonesia [53]. A subgroup analysis of the complications showed that patients with cerebral malaria had a higher mean lactate level than those with severe malaria without defining types of complications (6.23 vs. 4.60 mM). The results indicated that patients with cerebral malaria were likely to have increased blood lactate levels. This result supported the notion that the erythrocytes-infected trophozoite stage of *P*. *falciparum* consumed glucose and generated lactates as metabolites, and that the lactates moved from the plasma to the brain tissue or erythrocytes-infected *P*. *falciparum*-generated lactates in the brain [54]. Subgroup analyses of the different types of blood (whole blood or plasma) showed a higher pooled mean lactate level in plasma than in whole blood. This result supported that the plasma lactate concentration was higher than that of whole blood. This result was consistent with that of a previous study on blood lactate, which showed a higher lactate level in plasma than in whole blood [55]. Nevertheless, the heterogeneity of the pooled mean blood lactate in each subgroup remained high, indicating that the different types of blood samples were not able to explain the heterogeneity of the pooled mean blood lactate levels among the studies. In addition, a subgroup analysis conducted using an analyzer showed that a similar pooled mean blood lactate level was found in studies using both YSI and non-YSI analyzers. Nevertheless, the difference in blood lactate level found between studies using YSI and non-YSI analyzers was not directly analyzed. In addition, the heterogeneity of the pooled mean blood lactate in each subgroup remained high, indicating that the different types of analyzer used could not explain the heterogeneity of the pooled mean difference. The difference in different blood lactate analyzers was described by a previous study [56]. This previous study showed that YSI 23L gave a 22% lower result and YSI 1500 gave a 20% higher result than those obtained via the criterion/reference enzymatic method, indicating that there was some variability among analyzers [56].

The results showed a higher mean lactate level in patients with severe malaria than in those with uncomplicated malaria, as presented in all studies included in the meta-analysis [17,18,19,20,35,37,43,46,50]. There was a similar mean difference in lactate level between adults and children, as determined by a meta-regression of age. This result suggested that a high lactate level occurred similarly in all ages of patients with severe malaria. Nevertheless, there was a high heterogeneity in the pooled mean differences in lactate between the two groups. A higher mean difference in lactate level in children than in adults was demonstrated. In children, the highest mean difference in lactate between patients with severe malaria and those with uncomplicated malaria was demonstrated in the study conducted in Ghana between 1993 and 1995 [17]. In contrast, the lowest mean difference in lactate between the two groups was observed in the study conducted in Cameroon during 2007 [37]. The wide gap in the mean difference in lactate levels between the two studies might be due to the differences in the study sites, year of study, or study designs. In adults, the highest mean difference in lactate between patients with severe malaria and those with uncomplicated malaria was demonstrated in the study conducted in Bangladesh between 2005 and 2011 [20]. In contrast, the lowest mean difference in lactate between the two groups was observed in the study conducted in the Netherlands between 1999 and 2010 [50]. The wide gap in the mean difference in lactate levels between the two studies might be due to the differences in the characteristics of participants in the different study sites or years of study. However, the study conducted in Bangladesh enrolled patients with cerebral malaria; therefore, the difference in the mean difference between these two studies might be due to the characteristics of the participants enrolled. These results supported the notion that patients with cerebral malaria had a higher mean lactate level than those with severe malaria without cerebral malaria. Subgroup analyses of the different types of blood (whole blood or plasma) showed that there was a higher pooled mean difference in plasma or whole blood lactate level between patients with severe and patients with uncomplicated malaria. Nevertheless, a higher mean difference was found in studies measuring plasma lactate than in those measuring whole blood lactate. In addition, a subgroup analysis using analyzers showed a higher mean lactate level in patients with severe malaria than in those with uncomplicated malaria in studies using a YSI analyzer, but no difference in lactate level was found in studies using a non-YSI analyzer. This result might limit by the small number of studies conducted using the YSI analyzer in the subgroup or the variability among YSI analyzers [56].

The meta-analysis showed that patients with severe malaria who died had a higher mean difference in lactate levels than those with severe malaria who survived. Although the result presented with high heterogeneity, all studies included in the analysis presented a higher mean difference in lactate levels in patients with severe malaria who died compared to those with severe malaria who survived. This result indicated that lactate levels were that candidate marker for the risk of death among patients with severe malaria. In addition, the results suggested that the timely determination of plasma lactate upon admission may be helpful in the assessment of disease severity in travelers with imported malaria.

According to the WHO Guidelines for malaria issued in 2021, a plasma lactate level of more than 5 mmol/L was used as a marker for severe malaria [8]. For mortality, lactic acidosis in severe malaria was found to be strongly related to mortality [12,57]. Despite a recent study showing a strong risk factor for hyperlactatemia with a cutoff value of 5.2 mM for malaria-related death at 72 h [22], the present study showed that the pooled mean lactate in patients with severe malaria who died was 6.03 mM. Therefore, patients with severe malaria who had a blood lactate level above 6 mM are at a high risk of death. These findings confirm that hyperlactatemia is a candidate marker for mortality in patients with malaria. Improving lactate clearance during resuscitation may increase the survival of children living in malaria-endemic areas.

The present study had some limitations. First, there was high heterogeneity among the outcomes of the included studies. Therefore, the results of this meta-analysis need to be interpreted carefully. Second, only a limited number of included studies reported blood lactate levels for the groups of interest. Therefore, the results of the meta-analysis were dependent on the limited number of included studies. Third, the difference in mean lactate level between patients with cerebral malaria and other severe complications was varied and heterogeneous. This heterogeneity was limited by studies that did not report the lactate levels in patients with specific complications. Therefore, further studies are needed to investigate the difference in mean lactate level between patients with specific complications. Despite the fact that the WHO used plasma lactate as a criterion for severe malaria, the value of plasma lactate for predicting the risk of death remains unstudied. Further studies are needed to determine the significance of lactate-related deaths in an endemic malaria setting.

In conclusion, the present study showed the mean blood lactate level in patients with severe malaria. In addition, we found a high mean difference in blood lactate level between patients with severe malaria and patients with uncomplicated malaria. Furthermore, we found a high mean difference in blood lactate level between patients with severe malaria who died compared to those with severe malaria who survived. Further studies are needed to investigate the prognostic value of blood lactate levels for identifying patients with a high risk of developing severe malaria or death.

## Figures and Tables

**Table 1:** Characteristics of the included studies.

No.	Author (Publication Year)	Study Site (Year)	Study Design	Participants	Criteria for Severe Malaria	Number of Participants	Mean Age (Mean ± SD)	Age Groups (Years)	% Male	Lactate (mM), Mean ± SD	Types of Samples	Analyzer	Parasitemia (per uL), Mean ± SD
1.	Ackerman et al., 2021	Gambia, Malawi, Gabon, Kenya (2000–2005)	Prospective observational study	Children with severe malaria	NS	25,893	2.33 ± 0.78	<15	53.5	3.42 ± 1.17	Whole blood	Portable lactate analyzers (Arkay Lactate Pro LT-1710, YSI Corporation, USA in Kumasi)	80,961 ± 59,519
2.	Agbenyega et al., 1997	Ghana (1993–1995)	Case control study	Children with severe (54) and uncomplicated malaria (16)	WHO, 1990	70	Uncomplicated malaria: 5.25 ± 4.42, severe malaria: 3.5 ± 2.17	NS	NS	Uncomplicated malaria: 2.33 ± 0.86, severe malaria: 7.83 ± 4.66	Plasma	Lactate analyzers (YSI Instruments, Yellow Springs, Ohio, USA)	Uncomplicated malaria: 66,171 ± 125,068, severe malaria: 100,300 ± 519,459
3.	Agbenyega et al., 2003	Ghana (1997–1999)	Clinical trial	Children with severe malaria	NS	124	3.5 ± 3.7	1–10	50	7.45 ± 3.23 mg/dL	Whole blood	YSI Glucose Lactate Analyzer (Yellow Springs Instruments)	Unable to extract
4.	Akech et al., 2008	Kenya (2002–2005)	Prospective observational study	Children with severe malaria (and metabolic acidosis)	WHO, 2005	158	2.39 ± 0.6	NS	51.9	5.19 ± 4.04	NS	NS	At any density
5.	Barber et al., 2015	Malaysia (2010–2012)	Prospective observational study	Children with severe (21) and uncomplicated malaria (109)	Barber et al., 2015	130	Uncomplicated malaria: 26.5 ± 6.36, severe malaria: 32.5 ± 7.5	NS	NS	Uncomplicated malaria: 1.24 ± 0.3, severe malaria:1.92 ± 0.5	Whole blood	Bedside blood analysis (iSTAT system)	Uncomplicated malaria: 14,320 ± 8156, severe malaria: 92,913 ± 76,450
6.	Casals-Pascual et al., 2006	Kenya (NS)	Prospective observational study	Children with severe malaria (and severe anemia)	Molyneux et al., 1989	26	2.33 ± 1.05	NS	73	4.66 ± 2.57	Plasma	Lactate oxidase activity (Analox Instruments)	76,735 ± 84,098
7.	Conroy et al., 2019	Uganda (2008–2015)	Prospective cohort study	Children with cerebral malaria (and severe anemia)	The Ugandan Ministry of Health treatment guidelines	479	SMA (219): 3.1 ± 0.72, CM (260): 3.6 ± 0.72, total (479): 3.37 ± 0.76	18 months to 12 years	40.3	SMA (219): 5.15 ± 1.46, CM (260): 4.12 ± 1.32, total (479): 4.59 ± 1.48	NS	NS	SMA (219): 53,737.5 ± 36,038, CM (260): 97,517.5 ± 78,395.5, total (479): 77,501 ± 66,325
8.	Cserti-Gazdewich et al., 2013	Uganda (2007–2009)	Prospective observational study	Children with uncomplicated and severe malaria	WHO, 2010	1933	Uncomplicated malaria (1078): 3.12 ± 1.03, severe malaria (855): 1.95 ± 0.61, total (1933): 2.6 ± 1.04		53.1	Uncomplicated malaria (1052):2.25 ± 0.45, severe malaria (851): 5.65 ± 1.51, total (1903): 3.77 ± 1.99	Whole blood	Handheld lactate meter (LactatePro LT-1710; Arkray, Kyoto, Japan)	Uncomplicated malaria (1063): 96,250 ± 46,489, severe malaria (831): 116,750 ± 69,586, total (1894): 105,244 ± 58,644
9.	Cunnington et al., 2013	Gambia (2007–2011)	Case–control study nested within a larger prospective cohort study	Children with uncomplicated and severe malaria	WHO, 2000	423	Uncomplicated malaria (169): 6.49 ± 0.41, severe malaria (127): 4.25 ± 0.3, total (296): 5.52 ± 1.17	<16	Uncomplicated malaria: 55, severe malaria:59.8	Uncomplicated malaria (169): 2.1 ± 0.22, severe malaria (127): 4.71 ± 0.4, total (296): 3.21 ± 1.33	Whole blood	NS	Uncomplicated malaria (169):129,750 ± 24,200, severe malaria (127): 281,500 ± 37,260, total (296): 194,859 ± 81,159
10.	Day et al., 2000	Vietnam	Prospective cohort study	Adults with severe malaria	WHO, 1990	346	Deaths (52): 39.5 ± 15.2, Survived (294): 32.4 ± 13.7, Total (346): 33.5 ± 14.2	NS	77	Deaths (52): 4.35 ± 0.6, Survived (294): 3 ± 0.24, Total (154): 3.2 ± 0.56, Total (346): 3.2 ± 0.56	Plasma	Dedicated on-line analyzers (Analox, London, UK)	NS
11.	English et al., 1996	Kenya (NS)	Prospective observational study	Children with severe malaria	Molyneux et al., 1989	25	1.63 ± 0.83	0.33–3.08	56	9.75 ± 4.98 mmol/L	NS	Analox Instruments, London, United Kingdom	197,316 ± 214,305
12.	Inocent et al., 2009	Cameroon (2007)	Prospective observational study	Children with uncomplicated and cerebral malaria	WHO, 2000	139	Uncomplicated malaria (94): 3.46 ± 3.43, cerebral malaria (45): 2.29 ± 2.13, total (139): 3.08 ± 3.11 L	0–15	Uncomplicated malaria (94): 61.2, cerebral malaria (45): 48, total (139): 46	Uncomplicated malaria (94): 0.43 ± 0.03, cerebral malaria (45): 6.3 ± 0.56	NS	Spectrophotometry	Uncomplicated malaria (94): log10parasitemia 3.65 ± 0.03, cerebral malaria (45): 4.72 ± 0.79, total (139): 4 ± 0.67
13.	Ishioka et al., 2016	Bangladesh (2005–2011)	Prospective observational study	Adults with uncomplicated and severe malaria	Tran et al., 1996	286	Uncomplicated malaria (62): 31.1 ± 15.0, Severe and Deaths (70): 33.9 ± 13.7, Severe and Survived (154): 34.5 ± 15.4, Total severe (224): 34.3 ± 14.9		Uncomplicated malaria (62): 64.50, Severe and Deaths (70): 67.1, Severe	Uncomplicated malaria (62): 1.68 ± 0.33,Severe and Deaths (70): 6.43 ± 1.66, Severe and Survived (154): 3.5 ± 0.78, Total severe (224): 4.41 ± 1.76	Plasma	Olympus analyzer and a handheld automated analyzer (i-STAT, Abbott)	Uncomplicated malaria (62): 33,472 ± 20,014, Severe and Deaths (70): 112,883 ± 55,873, Severe and Survived (154): 118,472 ± 76,793, Total
									Survived (154): 77.9, Total severe (224): 74.5	severe (224): 116,725 ± 70,842			severe (224): 116,725 ± 70,842
14.	Ishioka et al., 2020	Bangladesh (2011–2016)	Prospective observational study	Adults with severe malaria	Tran et al., 1996	154	Deaths (41): 32 ± 4.62, Survived (111): 31 ± 4.63, Total (154): 31.3 ± 4.63	16–65	Deaths (41): 48.8, Survived (111): 69.9, Total (154): 64.3	Deaths (40): 5.78 ± 1.61, Survived (111): 2.95 ± 0.85, Total (154): 3.7 ± 1.67	Plasma	Handheld automated analyzer (i-STAT, Abbott)	Deaths (41): 95,927 ± 64,502, Survived (113): 72,085 ± 57,732, Total (154): 78,432 ± 60,326
15.	Jarvis et al., 2006	Gabon (1999–2000)	Prospective observational study	Children with severe malaria	Krishna et al., 2001	56	Severe malaria (56): 28.8 ± 5.78	1–10	Severe malaria (56): 52	Severe malaria (56): 3.95 ± 1.06	NS	YSI 2300 analyzer (Yellow Springs Instruments).	Severe malaria (56): 55,160
16.	Joice et al., 2016	Malawi (1996–2011)	Case control study	Children with cerebral malaria	Tayler et al., 2004	75	Cerebral malaria (75): 2.4 ± 0.55		Cerebral malaria (75): 51	Cerebral malaria (75): 11.2 ± 2.64	NS	NS	Cerebral malaria (75): 145,000 ± 120,114
17.	Krishna et al., 1994	Gambia (1988–1989)	Prospective observational study	Children with severe malaria	Molyneux et al., 1989	106	Severe malaria (106): 4.76 ± 2.17	1.5–18	NS	Severe malaria (106): 4.91 ± 3.08	NS	NS	Severe malaria (106): 383,400 ± 392,084
18.	Molyneux et al., 1989	Malawi (1987–1988)	Prospective observational study	Children with cerebral malaria	Molyneux et al., 1989	131	mean 3.6	<10	43.5	Deaths (20): 6.5 ± 4.8, Survived (99): 4.18 ± 2.5, Total (119): 4.57 ± 3.11	Plasma	YSI model, Clandon Scientific, Clandon Scientific, UK	Deaths (20): log parasitemia 5.56 ± 0.9, Survived (99): 5.17 ± 0.8, Total (119): 5.2 ± 0.82
19.	Newton et al., 2003	Thailand (1994–2001)	Prospective observational study	Adults with severe malaria	Hien et al., 1996	113	32.75 ± 14.7	>15	69.9	9.05 ± 7.82	Plasma	NS	208,320 ± 37,964
20.	O’Regan et al., 2016	Malawi (2008–2011)	Prospective observational study	Children with uncomplicated and cerebral malaria	NS	187	Uncomplicated malaria (52): 5.5 ± 0.3, cerebral malaria (135): 4.2 ± 0.1, total (187): 4.56 ± 0.61	6 months to 12 years	Uncomplicated malaria (52):51, cerebral malaria (135): 53, total (187): 54	Uncomplicated malaria (52): 2.5 ± 0.1, cerebral malaria (135): 6.9 ± 0.3, total (187): 5.67 ± 1.99	Plasma	NS	Uncomplicated malaria (52): 117,000 ± 30,000, cerebral malaria (135): 200,000 ± 25,000, total (187): 176,919.8 ± 45,688
21.	Olupot-Olupot et al., 2020	Uganda (2011–2012)	Prospective observational study	Children with severe malaria	WHO, 2000	662	Deaths (63): 1.54 ± 0.48, Survived (559): 1.65 ± 0.55, Total (119): 1.65 ± 0.55	2 months–12 years	Deaths (63): 61.9, Survived (559):51.2, Total (119): 57.7	Deaths (63): 5.78 ± 1.94, Survived (559): 2.37 ± 0.6, Total (119): 2.52 ± 0.71	NS	ARKRAY Factory, Shiga, Japan	Deaths (63): 41,390 ± 12,482, Survived (559): 61,110 ± 6322, Total (119): 58,800 ± 5744.8
22.	Ouma et al., 2020	Uganda (2008–2013)	Prospective cohort study	Children with severe malaria	Ouma et al., 2020	464	Cerebral malaria (253): 3.6 ± 0.72, severe anemia (211): 3.05 ± 0.74, total (464): 3.35 ± 0.72	NS	Cerebral malaria (253): 58.5, severe anemia (211):60.7, total (464): 59.5	Cerebral malaria (253): 4 ± 1.35, severe anemia (211): 4.97 ± 1.43, total (464): 4.45 ± 1.38	NS	NS	NS
23.	Planche et al., 2002	Gabon (1999–2000)	Prospective observational study	Children with uncomplicated and severe malaria	Planche et al., 2002	109	Uncomplicated malaria (23): 2.29 ± 0.48, severe malaria (86): 2.13 ± 0.43, total (109): 2.16 ± 0.45	NS	Uncomplicated malaria (23): 56.5, severe malaria (86): 48.8, total (109): 50.5	Uncomplicated malaria (23): 1.53 ± 0.29, severe malaria (86): 3.73 ± 01.04, total (109): 3.26 ± 1.3	Whole blood	YSI 2300 analyzer (YSI Instrument Corporation)	Uncomplicated malaria (23): 30,514.5 ± 14,075, severe malaria (86): 43,959.5 ± 29,876, total (109): 41,122 ± 27,806
24.	Sasi et al., 2007	Kenya (1996–1997, 2003–2004)	Case control study	Children with severe malaria	NS	61	2.83 ± 1.67	0.5–9.33	47	4.83	Whole blood	Analox lactate analyzer	NS
25.	Taylor et al., 1988	Malawi (1986–1987)	Prospective observational study	Children with altered consciousness	Teasdale et al., 1974	95	3.31 ± 2.15	0.75–8	54.7	4.7 ± 3.51	Plasma	YSI model, Clandon Scientific, UK	505,834 ± 442,801
26.	van Hensbroek et al., 1998	Gambia (1992–1994)	Prospective observational study	Children with cerebral malaria	Molyneux et al., 1989	452	4 ± 1.8	1–9	52	Cerebral malaria (452): 3.75 ± 1.31	NS	NS	Neurologic sequelae (20): 48,084, without neurologic sequelae (432): 46,238
27.	van Wolfswinkel et al., 2012	Netherlands (1999–2010)	Prospective observational study	Adults with uncomplicated and severe malaria	Anstey et al., 2007	141	Uncomplicated malaria (116): 40.5 ± 16.7, severe malaria (25): 2.85 ± 1.39, total (141): 45.3 ± 13.6	NS	Uncomplicated malaria (116): 79, severe malaria (25):60, total (97): 75.9	Uncomplicated malaria (116): 2.2 ± 1.45, severe malaria (25): 2.85 ± 1.39, total (141): 2.31 ± 1.46	Plasma	NS	Uncomplicated malaria (116): 50,479 ± 45,206, severe malaria (25): 497,052.5 ± 1,387,113, total (141): 129,658.6 ± 600,682
28.	Waller et al., 2016	Gambia (1985–1989)	Prospective observational study	Children with severe malaria	Molyneux et al., 1989	180	4.4 ± 2.0	NS	52.8	Deaths (27): 8.9 ± 5.9, Survived (153):4.7 ± 3.4, Total (180): 5.33 ± 4.14	Plasma	NS	Deaths (27): 425,383 ± 362,724, Survived (153): 428,138 ± 407,741, Total (180): 427,724 ± 400,358
29.	Watt et al., 2002	Thailand (NS)	Clinical trial	Adults with severe malaria	WHO, 2000	30	27.5 ± 9.36	18–50	100	4.32 ± 2.95	Serum	YSI lactate analyzer	267,515 ± 248,238
30.	Yeo et al., 2008	Indonesia (NS)	Prospective observational study	Adults with severe malaria	Hien et al., 1996	51	29 ± 11	>18		2.89 ± 0.5	NS	NS	35,067

Abbreviations: NS, not specified; SD, standard deviation.

## Data Availability

All data relating to the present study are available in this manuscript.

## Supplementary Materials

The following are available online at https://www.mdpi.com/article/10.3390/biology10111085/s1, Table S1: Search term, Table S2: Quality of the included studies, Checklist S1: PRISMA Checklist.
