# Supplementary material for: Alteration of Blood Lactate Levels in Severe Falciparum Malaria: A Systematic Review and Meta-Analysis
Source: Biology (Basel). 2021 Oct 22;10(11):1085. doi: 10.3390/biology10111085 (PMC8614809; doi:10.3390/biology10111085)
Supplement: Supplementary file 1 [file biology-10-01085-s001.zip › Table S1. Search term.pdf]

Alteration of blood lactate levels in severe falciparum malaria: A systematic review and meta-analysis

Polrat Wilairatana[1], Wanida Mala[2], Manas Kotepui[2], Kwuntida Uthaisar Kotepui[2*]

[1]Department of Clinical Tropical Medicine, Faculty of Tropical Medicine, Mahidol University, Bangkok, Thailand

[2]Medical Technology, School of Allied Health Sciences, Walailak University, Tha Sala, Nakhon Si Thammarat, Thailand

[*]**Corresponding author**

Manas Kotepui; manas.ko@wu.ac.th, Tel.: +66954392469

Polrat Wilairatana; polrat.wil@mahidol.ac.th

Wanida Mala; wanida.ma@wu.ac.th

Kwuntida Uthaisar Kotepui; kwuntida.ut@wu.ac.th

Table S1. Search term

**Databases**	**Search terms**	**Search date**
MEDLINE	(lactate OR "lactic acid") AND (malaria OR plasmodium) AND (severe OR complicated)	9 August 2021
Scopus	(lactate OR "lactic acid") AND (malaria OR plasmodium) AND (severe OR complicated)<br>Search option: All fields	9 August 2021

Web of Science	(lactate OR "lactic acid") AND (malaria OR plasmodium) AND (severe OR complicated)<br><br>Search option: All fields	9 August 2021
