# Supplementary material for: Alteration of Blood Lactate Levels in Severe Falciparum Malaria: A Systematic Review and Meta-Analysis
Source: Biology (Basel). 2021 Oct 22;10(11):1085. doi: 10.3390/biology10111085 (PMC8614809; doi:10.3390/biology10111085)
Supplement: Supplementary file 1 [file biology-10-01085-s001.zip › Table S2. Quality of the included studies.pdf]

Alteration of blood lactate levels in severe falciparum malaria: A systematic review and meta-analysis

Polrat Wilairatana[1], Wanida Mala[2], Manas Kotepui[2], Kwuntida Uthaisar Kotepui[2*]

[1]Department of Clinical Tropical Medicine, Faculty of Tropical Medicine, Mahidol University, Bangkok, Thailand

[2]Medical Technology, School of Allied Health Sciences, Walailak University, Tha Sala, Nakhon Si Thammarat, Thailand

[*]**Corresponding author**

Manas Kotepui; manas.ko@wu.ac.th, Tel.: +66954392469

Polrat Wilairatana; polrat.wil@mahidol.ac.th

Wanida Mala; wanida.ma@wu.ac.th

Kwuntida Uthaisar Kotepui; kwuntida.ut@wu.ac.th

Table S2. Quality the included studies

Observational studies: Cross sectional study

	Study	Score (out of 22)	Score (percentage)	Quality
1.	Ackerman et al., 2020	21	95	High
2.	Akech et al., 2008	19	86	High
3.	Barber et al., 2015	19	86	High
4.	Casals-Pascual et al., 2006	18	82	High

5.	Cserti-Gazdewich et al., 2013	21	95	High
6.	English et al., 1996	16	73	Low
7.	Inocent et al., 2009	17	77	High
8.	Ishioka et al., 2015	18	82	High
9.	Ishioka et al., 2020	19	86	High
10.	Jarvis et al., 2006	19	86	High
11.	Krishna et al., 1994	17	77	High
12.	Molyneux et al., 1989	18	82	High
13.	Newton et al., 2003	17	77	High
14.	O'Regan et al., 2016	16	73	Low
15.	Olupot-Olupot et al., 2020	20	91	High
16.	Planche et al., 2002	16	73	Low
17.	Taylor et al., 1988	16	73	Low
18.	van Hensbroek et al., 1998	18	82	High
19.	van Wolfswinkel et al., 2012	20	91	High
20.	Waller et al., 1995	17	77	High
21.	Yeo et al., 2008	18	82	High

Observational studies: Cohort studies

	Study	Score (out of 22)	Score (percentage)	Quality
1.	Conroy et al., 2019	21	95	High
2.	Cunnington et al., 2013	20	91	High
3.	Day et al., 2000	18	82	High
4.	Ouma et al., 2020	20	91	High

Case-control studies

	Study	Score (out of 22)	Score (percentage)	Quality
1.	Agbenyega et al., 1997	16	73	Low
2.	Joice et al., 2016	17	77	High
3.	Sasi et al., 2007	18	82	High

STROBE: Strengthening the Reporting of Observational Studies in Epidemiology
